# Uncovering Putative Bacterial Pathogens in Lakes in Aotearoa New Zealand Using Environmental DNA

**DOI:** 10.1002/ece3.72818

**Published:** 2026-02-11

**Authors:** Javier Atalah, Oliver Laroche, John K. Pearman, Susanna A. Wood, Marcus J. Vandergoes, Ian Davidson, Kate S. Hutson

**Affiliations:** ^1^ Cawthron Institute Nelson New Zealand; ^2^ Lincoln University Christchurch New Zealand; ^3^ GNS Science Lower Hutt New Zealand; ^4^ Centre for Sustainable Tropical Fisheries and Aquaculture, College of Science and Engineering James Cook University Townsville Australia

**Keywords:** aquatic diseases, bacteria, environmental DNA, lakes, metabarcoding, multiple stressors, surveillance

## Abstract

The emergence of aquatic diseases poses significant risks to ecological, social, cultural and economic values. Aquatic environments are intricately linked to human and animal health, as water can facilitate the spread and transmission of pathogens and waterborne diseases. We conducted an environmental DNA survey of 287 lakes across Aotearoa New Zealand, spanning a broad gradient of natural and human‐influenced conditions. Using a curated bacterial pathogens database, we detected 412 potentially pathogenic taxa—250 detected only in water and 162 found in both water and sediment; none were exclusive to sediment. Dominant groups included *Pseudomonas*, *Acinetobacter*, *Clostridium*, *Afipia* and Burkholderiaceae. Putative pathogens were ubiquitous in all lakes, including remote alpine sites with minimal human impact. Pathogen communities were associated with the extent of high‐productivity exotic grassland in the catchment and elevated nutrient levels, although richness was not linked to environmental drivers. Our findings show that eDNA can be a cost‐effective, broad‐spectrum screening tool that complements targeted diagnostics. The widespread occurrence of potential pathogens in lakes underscores the need for improved understanding of their ecological dynamics and the environmental conditions that promote disease outbreaks, supporting freshwater biosecurity, ecosystem management and public and animal health.

## Graphical abstract

Aquatic diseases threaten ecological, social, cultural and economic values. An eDNA survey of 287 lakes in Aotearoa New Zealand detected 412 potential pathogens. Key drivers of putative pathogen presence included high‐productivity exotic grassland and elevated nutrient levels.

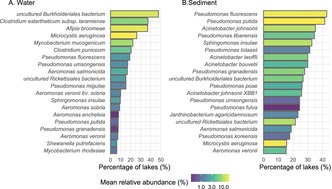

## 1 Introduction

The emergence of aquatic diseases poses significant risks to ecological, social, cultural and economic values associated with water bodies (Israngkura and Sae‐Hae 2002; Lane et al. 2022). Diseases caused by pathogens, like fungi, bacteria, viruses and parasites, can profoundly impact human and animal health (Behringer et al. 2020), leading to waterborne illnesses, economic losses in aquaculture, ecosystem disruptions and biodiversity declines (Johnson and Paull 2011; Peeler et al. 2007; Roberts et al. 2021). This threat is likely to be exacerbated by climate change and human‐induced stressors, including pesticide use and eutrophication (Byers 2021; Cascarano et al. 2021; French et al. 2022; Harvell et al. 1999; Samsing and Barnes 2024). Given the intricate link between aquatic environments and human and animal health, the transmission of pathogens through water is a pressing concern. Therefore, the One Health approach emphasises this interconnectedness, advocating for multidisciplinary collaboration across sectors to address and mitigate the risks posed by land management and emergent aquatic diseases (American Veterinary Medical Association 2008).

Lakes are essential aquatic ecosystems that contribute substantially to the health and well‐being of humans, animals and the environment. They provide invaluable resources and ecosystem services and hold significant cultural importance (Reynaud and Lanzanova 2017; Schallenberg et al. 2013; Sterner et al. 2020; Wood et al. 2023). For example, a substantial portion of the world's potable water is sourced from lakes, and they are also popular recreational destinations. Many lakes serve as vital habitats for species of ecological and cultural significance and support thriving fisheries (Schallenberg et al. 2013; Sterner et al. 2020). Unfortunately, lakes, like many aquatic systems worldwide, face significant pressure from various stressors, such as fluctuations in water levels, sedimentation, chemical and nutrient runoff, non‐native species, and the overarching effects of climate change (Pearman, Wood, et al. 2022; Thrush et al. 2011; Wood et al. 2023). Lakes, especially those close to densely populated areas, are highly vulnerable to stressors associated with changes in land use. These stressors can profoundly impact lake ecosystems, influencing ecological communities, including pathogens, their vectors and hosts (Eisenberg et al. 2007; Israngkura and Sae‐Hae 2002; Johnson and Paull 2011; Wu et al. 2016).

Understanding cost‐effective surveillance methods and factors driving pathogen emergence in aquatic ecosystems is vital for effective water quality management and disease prevention strategies. Surveillance enhances preparedness for emergent pathogen outbreaks, representing a key component of biosecurity systems (MacAulay et al. 2022). Typically, routine surveillance focuses on specific pathogens, employing techniques such as histopathology and necropsy of diseased hosts, bacteriology, or targeted molecular methods like PCR focused on species listed by the World Organisation of Aquatic Health (WOAH 2023). While these methods are often highly sensitive and specific, they cannot screen for a wide range of potential emergent diseases. Environmental DNA (eDNA) offers a promising alternative for cost‐effective screening of a broad range of pathogens (Amarasiri et al. 2021; Davis et al. 2021; Sieber et al. 2020) and is increasingly used for monitoring aquatic health, representing a dynamic multipurpose surveillance approach (Gomes et al. 2017; Sieber et al. 2024). Although numerous studies have employed eDNA and other molecular tools to detect pathogens from diverse hosts and various aquatic environments (Amarasiri et al. 2021; Farrell et al. 2019; Huver et al. 2015; Sieber et al. 2024), none have specifically used eDNA as an approach to assess bacterial pathogen risk in lakes. While the presence of pathogens doesn't always lead to disease, due to the complex interplay of pathogen, environment and host susceptibility (Engering et al. 2013; Hutson et al. 2023; Samsing and Barnes 2024), detecting them is the first step in evaluating potential risk. Given the scarcity of data on a wide diversity of aquatic pathogens, eDNA metabarcoding offers a valuable tool for assessing the presence of opportunistic organisms with a propensity to cause disease under certain environmental scenarios.

We used a national‐scale sampling programme of lakes to assess bacterial pathogen prevalence. Aotearoa New Zealand has more than 3800 lakes, and nearly half are in a degraded ecological state, primarily due to nutrient inputs (Wood et al. 2023). No studies have investigated the diversity, prevalence and main drivers of bacterial pathogen communities in these lakes. The objectives of this study were: (1) To assess the potential of eDNA as a surveillance tool for putative bacterial pathogens within two distinct ecological niches: the water column and sediment; (2) To elucidate key anthropogenic and ecological drivers, such as land use and nutrient concentrations, that govern the distribution and abundance of waterborne bacterial pathogens in these two habitats; and (3) To assess the predictive power of these identified drivers to identify putative bacterial pathogen occurrence. Ultimately, the study aimed to improve approaches to investigating emerging aquatic diseases, including developing novel detection and predictive tools.

## 2 Methods

### 2.1 Study Lakes and Sampling

**FIGURE 1:**
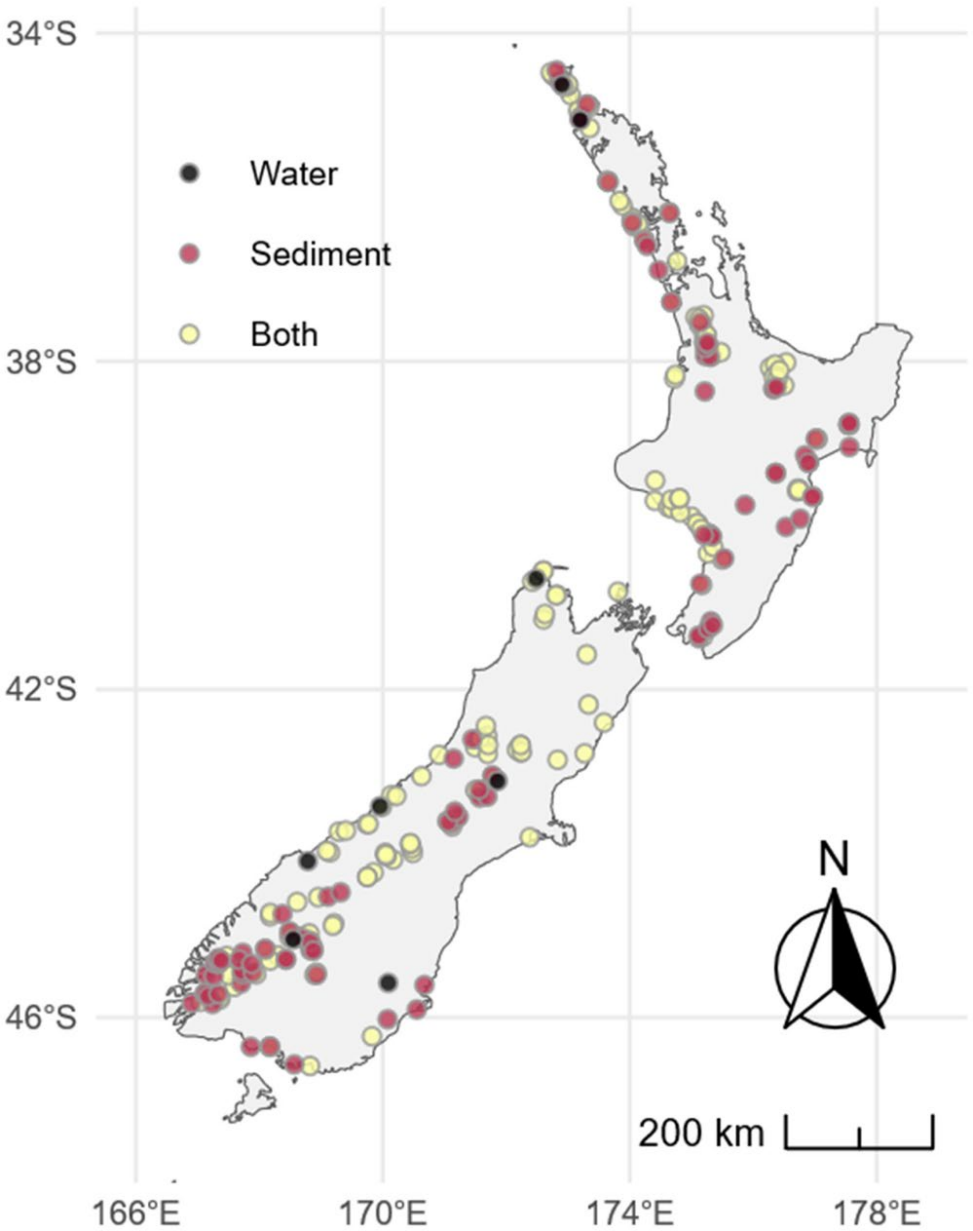
Map of Aotearoa New Zealand, showing the lakes where sediment and water samples were collected to detect bacterial pathogens using environmental DNA metabarcoding.

**FIGURE 2:**
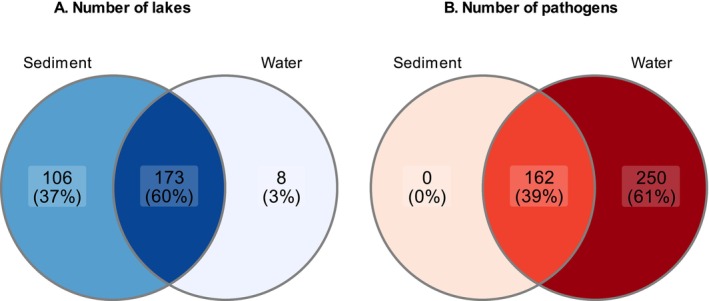
Venn diagrams showing: (A) the number of sampled lakes and (B) the number of pathogenic bacterial taxa detected in sediment and water samples. The intersection of sets represents shared lakes and taxa, respectively.

A total of 287 lakes in Aotearoa New Zealand were sampled (Figure 1) between September 2018 and November 2020 as part of the Lakes380 research programme (lakes380.com). Each lake was sampled once during this period, providing a broad spatial snapshot rather than capturing seasonal variation. Sediment samples were collected from 279 lakes. Water samples were collected from 181 lakes, and 173 were sampled for both (Figure 2A). Lakes were carefully selected to encompass various environmental gradients (see details in Wood et al. 2023), including trophic status, altitude, size, depth and catchment characteristics (Supporting Information S1). Altitude and lake depth were assessed in situ at each lake. Satellite imagery from the Land Cover Database Version 5 (Land Care Research Ltd 2020) was used to derive seven primary land cover variables: (1) Native vegetation, (2) Urban, (3) Non‐native vegetation, (4) Forestry, (5) High Production Grassland (HPG; representing the proportion of high‐productivity grassland, including exotic grasslands used for wool, lamb, beef, or dairy), (6) low productivity grassland (LPG; agricultural grassland with low stock density) and (7) Other. The lakes were classified into six trophic statuses: microtrophic, oligotrophic, mesotrophic, eutrophic, supertrophic and hypertrophic, based on the sediment bacterial trophic index (Pearman, Wood, et al. 2022).

Lake sediment sampling methodology followed that described by Pearman, Wood, et al. (2022). Triplicate sediment samples were collected using a Ponar grab (sampling area 0.023 m^2^; Wildco, USA) at the deepest part of the lake. Samples were collected from shallower bays for five lakes exceeding a depth of 100 m. A subsample of approximately 1 g of undisturbed sediment was taken from the surface layer (top 5 mm) using a sterile spatula. These samples were then placed in LifeGuard Soil Preservation Solution (Qiagen, Germany) and stored at −20°C for subsequent DNA extraction. Secchi Disk depth was recorded for all lakes.

Water samples were collected following the methodology described by Pearman et al. (2023). Briefly, triplicate surface water samples were collected in 500 mL bottles. The bottles were sterile and washed three times with lake water before the sample was taken. For each replicate, 100 mL was filtered through a 0.22 μm S‐Pak filter (Millipore Sigma, Burlington, MA, USA) and stored at −20°C for subsequent DNA extraction.

Sediment total nitrogen, total phosphorus, total dissolved carbon and chlorophyll‐a were measured as described in Waters et al. (2023). Surface water for chlorophyll‐a was analysed following the APHA 10200 H method at Watercare Laboratories (Auckland, New Zealand). The APHA 4500 method was used on a flow injection analyser to measure total nitrogen and total phosphorus. Dissolved organic carbon analysis was undertaken by combustion analysis at 850°C using APHA 5310 B methods. For detailed procedures, see Pearman et al. (2023) and Waters et al. (2023).

DNA from water and sediment samples was extracted using the DNeasy PowerSoil Kit (Qiagen, Hilden, Germany). The water filters were cut into pieces and placed in the PowerBead tubes of the kit, while for the sediment, a 0.25 g subsample was used. All further steps followed the instructions of the DNeasy PowerSoil Kit using a QIAcube. Negative extraction controls, consisting of the extraction buffer, were included after every 23 samples. The bacterial 16S rRNA gene was amplified as described in Pearman et al. (2023), targeting the V3–V4 region (~469 bp) using the primers 341F and 805R (Klindworth et al. 2013) with Illumina overhang adapters (Kozich et al. 2013). This primer pair was selected because it is widely used for bacterial community profiling, offers broad taxonomic coverage, and aligns with existing Lakes380 sequencing data, enabling a cost‐effective large‐scale survey across hundreds of lakes. PCR reactions were undertaken in triplicate. Cleaning and normalisation were undertaken using SequalPrep Normalisation plates (Thermo Fisher Scientific, Waltham, USA). Sequence libraries were constructed following the Illumina 16S metagenomic library prep manual and sequenced on an Illumina MiSeq. Raw reads are stored in the NCBI short read archive under the accession numbers PRJNA762383, PRJNA606991, PRJNA750120 and PRJNA813318, and ecological patterns have been previously published (Pearman, Thomson‐Laing, et al. 2022).

### 2.2 Molecular Analyses, Bioinformatics and Assignment of Putative Pathogens

Bioinformatics and molecular analyses are described in Pearman et al. (2020). Briefly, primers were removed from the raw reads using cutadapt, allowing a single mismatch (Martin 2011). To infer amplicon sequence variants (ASVs), the DADA2 package (Callahan et al. 2016) was used within the software R (R Core Team 2023). Taxonomic classification was undertaken against the SILVA 138 (Pruesse et al. 2007) using the RDP classifier (Wang et al. 2007) and the *assignTaxonomy* function of DADA2, to attribute taxonomy down to genus level, and the *assignSpecies* function, which performs exact matching to assign species following recommendations from Edgar (2018). In case of multiple exact matches, species names were concatenated to retain all information.

We used a comprehensive, pre‐defined database for bacterial pathogens (Yang et al. 2023) to categorise identified bacterial species as potential pathogens based on bioinformatic taxonomic assignment. This curated reference, part of the ‘One Health’ vision, enabled efficient identification of animal, plant, and zoonotic pathogens in biological and environmental samples and was crucial for distinguishing closely related non‐pathogenic strains. Pathogen ASVs were filtered from the whole bacterial community dataset based on the multiple bacterial pathogen detection pipeline and database provided by Yang et al. (2023) and publicly available at https://github.com/LorMeBioAI/MBPD. Briefly, ASVs were queried against the database using *vsearch* (Rognes et al. 2016; version 2.22.1), following parameters described in Yang et al. (2023). Only hits with 100% similarity were considered putative pathogens and retained, hereafter termed ‘pathogens’. This threshold was selected based on Edgar (2018) to minimise false positives. It is also worth noting that both the Silva v138 species database and MBPD database contain strain‐level sequence data, reducing the chances of missing assignations (false negatives) based on intra and intergenomic variation. Identified pathogens included bacteria affecting animals, humans and plants, as well as opportunists responsible for zoonotic diseases. Several sequences in the database are labelled as ‘uncultured’ taxa, originating from environmental samples that have not been grown under laboratory conditions. These sequences do not match any known taxa, so they could represent new, undescribed species. As a result of this filtering process, 1,031,098 and 158,136 bacterial ASVs from sediment and water samples, respectively, were classified as environmental bacteria rather than putative pathogens and excluded from further analysis.

### 2.3 Statistical Analyses

A one‐way ANOVA was performed to compare pathogen richness across trophic levels in both water and sediment samples, followed by Tukey post hoc pairwise comparisons. The filtered community data were transformed into relative abundance after removing non‐pathogenic taxa by dividing the number of reads by the total number of reads in the sample. Distance‐based redundancy analysis (Legendre and Anderson 1999) assessed the relationship between bacterial pathogen communities and 14 environmental drivers relevant to this dataset (catchment characteristics and physiochemical variables; Supporting Information S1) using the *vegan* package (Oksanen et al. 2018). Ecological drivers were first scaled, centred and Yeo‐Johnson transformed before the analyses to improve their normality and comparability. Models were fitted based on Jaccard's dissimilarities of community data, with all environmental drivers as predictors (Supporting Information S1). Models were tested using a permutational analysis of variance, beginning with a marginal test where individual variables were fitted separately to assess their relationship with pathogen community data. The relationship between the number of pathogens per lake and environmental variables was tested using generalised models with negative binomial errors to account for overdispersion. Models were validated by inspecting simulated residuals using the *DHARMa* package (Hartig 2022).

## 3 Results

A total of 412 bacterial pathogens were detected in lake sediment and water samples (Supporting Information S2), with 250 exclusively detected in water samples and 162 detected in both sediment and water samples (Figure 2). No pathogens were unique to sediment samples. The average number of pathogen taxa per lake was significantly higher in the water column (25.9 taxa ± 1.7 SE) compared to sediment (5.98 ± 0.4, *p* < 0.001). On average, pathogens represented 0.2% (±0.6 SD) of the total bacterial community in sediment samples and 2.4% (±2.1%) in water samples (Supporting Information S3).

**FIGURE 3:**
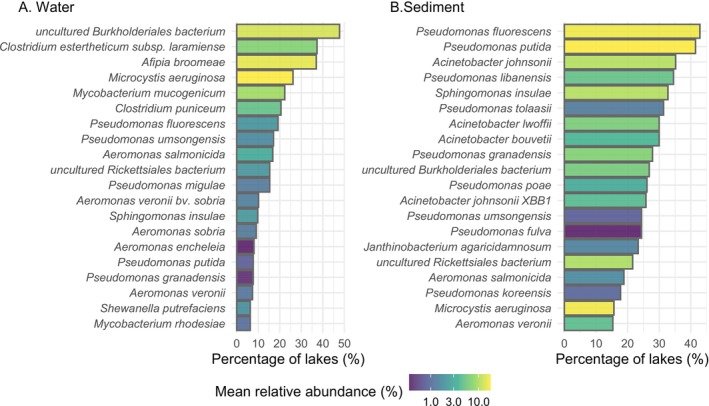
Percentage of lakes where the most common putative bacterial pathogen taxa (sequence identity matches) were detected in water (A) and sediment (B) samples. Bars are coloured based on the mean relative abundance, calculated after removing non‐pathogenic taxa (percentage of the total number of reads in the sample) across all lakes.

The prevalence of each pathogen in the sediment and water column was low, occurring at a median of three and four lakes, respectively (Supporting Information S4). The most prevalent pathogens in the water column were *Pseudomonas fluorescens* , detected in 123 lakes, followed by *Pseudomonas putida* in 119 lakes, *Acinetobacter johnsonii* in 101 lakes, *Pseudomonas libanensis* in 99 lakes and *Sphingomonas insulae* in 94 lakes (Figure 3). Among the pathogens found in lake sediments, the most prevalent was an uncultured *Burkholderiales bacterium*, detected in 137 lakes, followed by *Clostridium estertheticum sub* sp. *laramiense* in 107 lakes, *Afipia broomeae* in 106 lakes, *Microcystis aeruginosa* in 75 lakes and *Mycobacterium mucogenicum* in 64 lakes (Figure 3).

**FIGURE 4:**
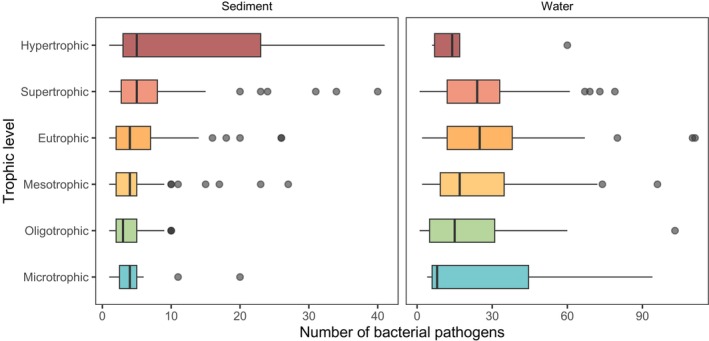
Boxplot of the number of bacterial pathogens per lake by trophic status in sediment and water samples.

The number of sediment pathogen taxa per lake ranged between one and 41, with a median of four. For water samples, pathogen richness was higher, with a median of 20, ranging between one and 181 bacterial pathogens per lake. There was a marginally significant effect of altitude on the number of pathogens in water samples and altitude and latitude on the number of pathogens in sediment samples (*p* < 0.05, Supporting Information S5). No other significant effects were detected for the other 12 predictor variables tested. The median number of pathogens in sediment and water varied marginally between trophic levels (Figure 4, *p* = 0.04), but no pairwise differences were found.

**FIGURE 5:**
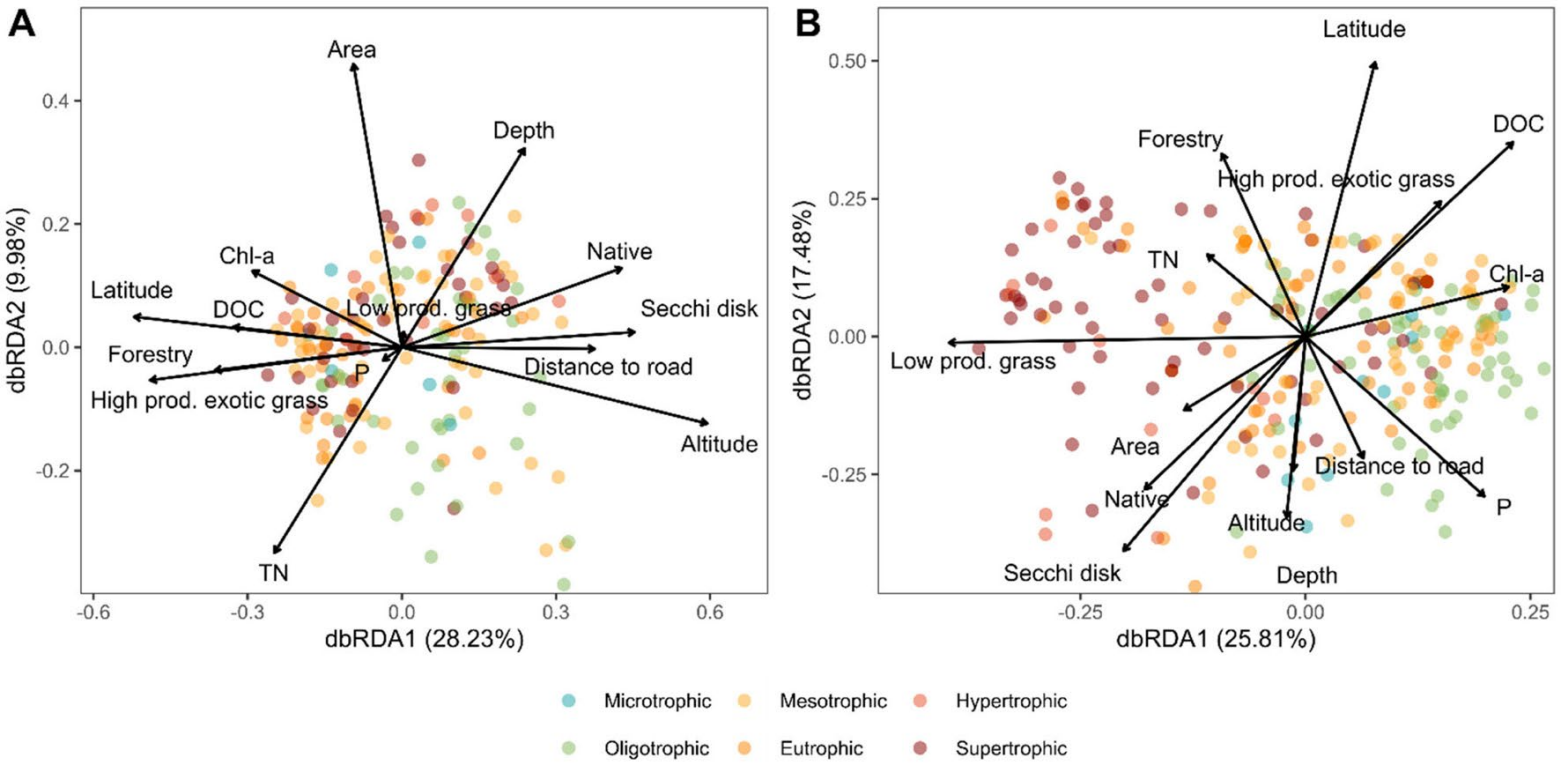
Distance‐based redundancy analysis (dbRDA) biplot of environmental drivers and bacterial pathogen communities in: (A) water and (B) sediment of lakes with varying trophic status. TN: Total Nitrogen, P: Total Phosphorus, High prod. Exotic grass: High productivity grassland, Low prod. Grass: Low productivity grassland, Area: Lake area, Chl‐a: Chlorophyll‐a, DOC: Dissolved organic carbon. See Supporting Information S1 for details on environmental drivers.

Distance‐based redundancy analysis (dbRDA) revealed that all predictor variables together explained only 9% of the total variation in water‐column pathogen community data. The most significant driver was high‐productivity exotic grassland (*p* < 0.001, Supporting Information S5), followed by total nitrogen, which had a marginally significant effect (*p* = 0.05, Supporting Information S6). All other variables had no significant impact on the structure of the water column pathogen communities. Water column pathogen communities were separated by the effect of high‐productivity exotic grassland along the first axis (28.23%; Figure 5A). This axis negatively correlates with forestry, latitude, dissolved organic carbon and chlorophyll‐a. In contrast, it positively correlates with native cover, distance to roads, altitude and Secchi disk depth along the opposite end of the first axis (Figure 5A).

The dbRDA for sediment bacteria explained 6% of the total variability in the data. The most significant drivers structuring pathogens were phosphorus (*p* < 0.01), low‐productivity grassland (*p* < 0.05) and latitude (*p* < 0.05). Sediment pathogens were separated along the first dbRDA axis (25.81%) with low‐productivity grassland, negatively correlated with total nitrogen, lake area and native cover, and positively associated primarily with phosphorus and chlorophyll‐a (Figure 5B).

## 4 Discussion

A total of 412 potentially pathogenic bacteria that can impact animal and human health were detected in the water column and sediment of nearly 300 lakes across Aotearoa New Zealand. These lakes cover a diverse range of gradients in natural characteristics and degrees of human impact. Surprisingly, at least one pathogen taxon was detected in the water column or the sediment in all lakes, including remote alpine lakes in the South Island. Of all detected pathogens, 60% were exclusively found in water samples, while the remainder appeared in sediment and water samples. Interestingly, no unique sediment pathogens were detected. Communities appear to be shaped to some degree by the extent of high‐productivity exotic grassland in the catchment and nutrient concentrations. However, the predictive power of our models was limited. This research highlights the potential of using eDNA as a surveillance tool for potentially pathogenic bacteria in lakes, for example as part of routine monitoring by regional authorities (Land, Air, Water Aotearoa 2024). It also demonstrates the role of human‐driven factors in shaping pathogen communities and indicates challenges in forecasting these pathogens' occurrences. Multivariate analysis and modelling suggest lake pathogen distributions are not solely associated with anthropogenic vectors or direct human activities, indicating other significant mechanisms of introduction and connection among waterbodies, such as wildlife (Johnson and Paull 2011; Murray 2009).

Modelling revealed that the primary (but still relatively weak) drivers of pathogen communities in aquatic ecosystems are the extent of high‐productivity exotic grassland in the catchment area and nutrient concentrations. The relationship with intensive grazing management highlights the pressures different land uses exert on ecosystem functioning, including shaping pathogen diversity and distribution (Lenaker et al. 2017; Wilkes et al. 2011). Elevated levels of nutrients, such as nitrogen and phosphorus, typically linked to high‐intensity agriculture, trigger profound changes in lake functioning, such as eutrophication and associated algal growth (Abell et al. 2011; Wood et al. 2023). This, in turn, leads to alterations in microbial communities, including bacterial pathogens (Pearman, Wood, et al. 2022). Our results highlight the intricate relationship between land use, human activities and lake microbial ecology. They also emphasise the need for effective ecosystem management strategies to mitigate pathogen‐related risks. These identified stressors have previously been acknowledged as relatively weak drivers of bacterial communities in lakes (Pearman et al. 2023). This consistency across microbial groups suggests common underlying mechanisms governing community dynamics in response to environmental changes.

While our results highlight human‐driven factors shaping pathogen communities, they also stress the challenges in forecasting their occurrences. In this and previous related studies (Pearman, Thomson‐Laing, et al. 2022), a small amount of variation in bacterial communities was accounted for in the models, highlighting the need to consider other drivers, such as natural and human transmission vectors. The model's predictive power was limited at both the community and single taxa levels, possibly reflecting complex interactions between the environment, hosts and pathogens that govern the occurrence of these organisms. The relatively low explanatory power of the dbRDA further suggests that temporal variability (sampling spanned multiple seasons and years) and interactions between land‐use, climate, and unmeasured local factors likely contributed to unexplained variation. Although taxa richness in sediment generally increased with trophic levels, this pattern was less evident in water samples, and the absence of significant pairwise differences indicates that pathogen presence is broadly distributed rather than strongly structured across trophic levels. Some oligotrophic lakes had high pathogen richness values. Three of these were large glacial lakes, which are exposed to relatively high levels of human activity, particularly in summer, due to tourism. A logical explanation was less apparent for remote high alpine lakes. In these instances, the presence of pathogens could be related to introduced terrestrial and aquatic organisms (e.g., geese, non‐native fish, deer and other browsing mammals) that can act as vectors (Johnson and Paull 2011; Murray 2009). The findings of the present study highlight the limited understanding of critical epidemiological factors associated with bacterial pathogens, including transmission modes, reservoirs, vectors and stability. Furthermore, they underscore the significant turnover among lakes within a diverse national‐scale community, which can introduce variability that is challenging to capture with statistical models. Pathogen distribution was highly skewed, with a notable prevalence of rare taxa, which can introduce additional variability and pose challenges for prediction. This dominance of rare taxa likely reflects a combination of ecological processes, including dispersal limitations, environmental filtering, and stochastic dynamics, which shape microbial community structure. High levels of richness driven by relatively rare species also provide a wide scope for many taxa to emerge as aquatic health problems in future, particularly if environmental conditions shift or host susceptibility changes, posing challenges for managing outbreaks of emerging disease issues.

Many of the bacteria detected in the present study are opportunistic pathogens that may be capable of causing disease that significantly impacts animal health. There was widespread presence of opportunistic pathogenic bacteria such as *Pseudomonas*, *Acinetobacter* and *Sphingomonas* within the sediment. In contrast, taxa, including *Burkholderiales*, *Clostridium*, *Afipia* and *Microcystis*, were in the water column. These organisms pose significant risks to animal and human health and can spread through water transmission or direct contact with infected individuals. For example, *Pseudomonas* is a common bacteria group that can cause a range of public health threats, from skin and eye infections to serious life‐threatening illnesses, particularly in immunocompromised people (Mena and Gerba 2009). *Acinetobacter* is largely an opportunistic pathogen that is known to cause infections among marine and freshwater fish with severe impacts on wild populations and aquaculture industries (Bi et al. 2023). *Microcystis*, although not an infectious pathogen, is a cyanobacterium known for forming harmful algal blooms. It can also produce toxins that pose chronic and acute human and animal health risks (Harke et al. 2016; Wood et al. 2016).

Positive eDNA results provide initial evidence of the potential presence of a pathogen in the environment. For example, our eDNA screening revealed the presence of *Aeromonas* spp. (the associated ASV matched 100% with *A. salmonicida* , *A. hydrophilia* and *A. enterica* with 100% similarity). Both typical and atypical *A. salmonicida* are legally notifiable in New Zealand, featuring prominently on the priority list of potential disease‐causing organisms (Ministry for Primary Industries 2024). *Aeromonas salmonicida* subspecies *salmonicida* is the causative agent of furunculosis, a disease characterised by boil‐like lesions and bleeding in salmon and trout, often proving fatal to infected fish within 2–5 days (Wiklund and Dalsgaard 1998). The disease furunculosis has not been reported in Australia or New Zealand. An atypical *Aeromonas salmonicida* was considered an incidental finding during a disease investigation into lamprey reddening syndrome in rivers in Aotearoa New Zealand (Brosnahan et al. 2019). *Aeromonas salmonicida* can survive for several months outside its fish host, thriving in marine and freshwater environments. The target region in this study was unable to resolve species affiliation in this genus. The definitive confirmation of the presence of the subspecies requires the application of the Australian and New Zealand standard diagnostic procedure (Buller et al. 2021). In this context, the proposed screening tool has proven helpful in alerting managers and stakeholders about the potential occurrence of a notifiable organism and guiding subsequent targeted surveillance efforts.

Surprisingly, our investigation did not identify any pathogens present in lake sediment samples alone. Although the most common bacterial pathogens from water samples mirrored those in the sediment, less common and rare ones were only found in water samples. Any bacterial species that occur in sediments are presumed to have entered that habitat via the water column, so the sequential ‘infection’ of habitat types may help explain the lack of unique bacterial taxa in sediments. Most previous studies have used water samples when targeting pathogens using eDNA, but have also confirmed the presence and prevalence in sediment samples (Holman et al. 2019). eDNA is typically more concentrated in sediment than in water samples (MacAulay et al. 2022); for instance, Asian carp DNA was found to be 8–1800 times more concentrated in sediment than in water (Turner et al. 2015). These findings emphasise the role of lake sediments as both a reservoir and a long‐term archive of bacterial pathogen DNA. In contrast, pathogens detected in the water column appear more transient. This difference highlights the distinct nature of pathogen persistence between lake sediments and the water column. For instance, sediment samples can be valuable for obtaining time‐integrated estimates of pathogenic risks or for elucidating paleoecological drivers (Gregersen et al. 2023; Picard et al. 2022; Thomson‐Laing et al. 2024). Water samples are likely more relevant for real‐time screening or more immediate aquatic health concerns.

### 4.1 eDNA and Pathogen Screening

Ensuring cost‐effective detection tools for aquatic bacterial pathogens is a critical step in averting their emergence and spread and mitigating their impact. Our study highlights the advantages of analysing eDNA collected from environmental samples instead of animal sampling as a cost‐effective, non‐destructive screening tool. This approach is particularly beneficial for accessing aquatic bacterial pathogens in environments which are challenging to sample using traditional approaches or when targeting organisms associated with difficult‐to‐collect wild animals. Environmental DNA‐based sampling and analysis is a time‐ and cost‐effective screening approach that does not use sacrificial hosts needed for individual animal sampling (Rusch et al. 2018). However, this approach also has limitations. For example, metabarcoding specificity is often limited (Sieber et al. 2020), and its limited resolution for confirming species‐level identification might lead to false positives. Moreover, eDNA detection does not confirm organism viability, virulence, or disease risk, as many taxa are opportunistic and their pathogenicity depends on environmental context and host susceptibility. Before implementing any management measures, it is imperative to confirm the presence and potential activity of a potential pathogen using targeted assays such as bacteriological cultures, PCR, metagenomics, or toxin analyses. Alternative molecular approaches, such as long‐read sequencing of the full 16S rRNA gene (e.g., Oxford Nanopore or PacBio) or shotgun metagenomics, could improve taxonomic resolution and strain‐level identification in future studies. However, these approaches are currently less practical for large‐scale surveys due to higher costs, lower sensitivity for rare taxa, and substantial data‐processing requirements in the case of shotgun metagenomics. In this context, we emphasise the role of eDNA metabarcoding in early‐stage pathogen screening while acknowledging the need for complementary methods to enhance our understanding of pathogen dynamics. It is also important to note that the presence of bacterial pathogens does not necessarily translate into the manifestation of a disease in an infected host. Disease occurs from a complex interaction between the pathogenic agent, suitable environmental conditions, and susceptibility of the host organism (Engering et al. 2013; Hutson et al. 2023). As such, according to the World Organisation for Animal Health, data obtained from eDNA methods are unsuitable to support declarations of freedom from listed diseases (WOAH 2022).

When considering a monitoring programme or sampling events, water quality can influence the efficacy of eDNA sampling, and the acidity of lake water can accelerate DNA degradation (Seymour et al. 2018). Sediment sampling is more demanding, expensive, and logistically complex than water sampling. For instance, water samples can be collected from the shoreline, from a boat, or using drones or automated buoys. Sediment sampling necessitates using cores, benthic grabs, divers, or ROVs, which tend to be more expensive. Ultimately, the decision to use water over sediment samples for pathogen surveillance will depend on the objectives of the study. Based on our findings, which suggest that water samples capture a broader diversity of pathogens, including rare taxa, it is advisable to prioritise water sampling. This method has the potential to provide a cost‐effective tool for identifying prevailing pathogenic threats in lakes.

Globally, aquatic disease databases and systematic monitoring programmes are generally scarce. This translates into inadequate knowledge and high uncertainty of geographic ranges for aquatic pathogens, making it challenging to predict their occurrence and emergence in response to environmental and climate change. In Aotearoa, New Zealand, reports of suspected exotic or emerging aquatic pathogens are investigated by the Ministry for Primary Industries. There are also ongoing targeted surveillance programmes. General surveillance by citizens is operationalised when they report suspected pests and diseases through a hotline system. Received notifications are screened and investigated by aquatic health staff, and if further research is needed, the cases are sent to an investigation and diagnostic centre. In addition, 13 specialised surveillance programmes target specific biosecurity risks (Ministry for Primary Industries 2024). As eDNA is increasingly adopted as part of routine monitoring of aquatic systems, careful consideration is needed to ensure eDNA technologies are appropriate for the surveillance objective, selected pathogen and specific environment (WOAH 2022). With further validation, eDNA could become a useful pathogen screening tool, especially for rare, valuable or difficult‐to‐sample susceptible hosts. The interpretation and implication of eDNA results need to be considered prior to testing, especially when legally notifiable or WOAH‐listed pathogens may be indicated. It should be clear when further actions (e.g., notification of authorities, direct sampling of susceptible species) are required. Expert‐curated databases of bacterial sequences associated with animal and human pathogens would facilitate this process. Our study was greatly facilitated by a comprehensive, already‐defined database for bacterial pathogens (Yang et al. 2023). Further development and expansion are required to incorporate other taxonomic groups, such as eukaryotic pathogens. Such a database could lead to the creation of a screening tool to understand changes in pathogen prevalence and distribution, which would assist in informing risk analyses, aquatic health management and robust biosecurity systems.

## Author Contributions

**Javier Atalah:** conceptualization (equal), data curation (equal), formal analysis (equal), funding acquisition (equal), investigation (equal), methodology (equal), project administration (equal), writing – original draft (equal), writing – review and editing (equal). **Oliver Laroche:** data curation (equal), formal analysis (equal), methodology (equal), writing – review and editing (equal). **John K. Pearman:** data curation (equal), formal analysis (equal), methodology (equal), writing – review and editing (equal). **Susanna A. Wood:** conceptualization (equal), data curation (equal), funding acquisition (equal), investigation (equal), methodology (equal), project administration (equal), writing – review and editing (equal). **Marcus J. Vandergoes:** conceptualization (equal), data curation (equal), funding acquisition (equal), investigation (equal), methodology (equal), project administration (equal), resources (equal), writing – review and editing (equal). **Ian Davidson:** funding acquisition (equal), project administration (equal), writing – review and editing (equal). **Kate S. Hutson:** funding acquisition (equal), project administration (equal), writing – review and editing (equal).

## Conflicts of Interest

The authors declare no conflicts of interest.

## Supporting information


**Data S1:** ece372818‐sup‐0001‐SupplementaryMaterials.docx.

## Data Availability

The scripts and data used for the analyses are available at https://github.com/jatalah/EAD_LAkes380_pathogens.
