# Supplementary material for: Uncovering Putative Bacterial Pathogens in Lakes in Aotearoa New Zealand Using Environmental DNA
Source: Ecol Evol. 2026 Feb 11;16(2):e72818. doi: 10.1002/ece3.72818 (PMC12892091; doi:10.1002/ece3.72818)
Supplement: Supplementary file 1 — Data S1: ece372818‐sup‐0001‐SupplementaryMaterials.docx. [file ECE3-16-e72818-s001.docx]

**SUPPLEMENTARY MATERIAL**

Supplementary Material 1. Summary statistics (mean, standard deviation, minimum, 25th percentile, median, 75th percentile, maximum) of lake catchment characteristics and physicochemical variables used as predictor variables for bacterial pathogen communities.

|  |  | Mean | S.D. | Min. | p25 | Median | p75 | Maximum |
| --- | --- | --- | --- | --- | --- | --- | --- | --- |
| Total Nitrogen (mg m^-3^) | 81 | 1.09 | 0.74 | 0.06 | 0.58 | 0.91 | 1.6 | 3.3 |
| Total Phosphorus (mg m^-3^) | 75 | 1518.15 | 934.1 | 130 | 907.5 | 1295 | 1812.5 | 5430 |
| High productivity grassland (% catchment) | 0 | 34.62 | 37.36 | 0 | 0 | 14.96 | 71.02 | 100 |
| Low productivity grassland (% catchment) | 0 | 7.02 | 17.33 | 0 | 0 | 0 | 2.86 | 100 |
| Latitude (decimal degree) | 0 | -41.28 | 3.53 | -46.59 | -44.53 | -42.18 | -38.2 | -34.45 |
| Altitude (m) | 2 | 389.94 | 467.4 | 0 | 38 | 171 | 674 | 1839 |
| Lake area (ha) | 10 | 647.65 | 3382.95 | 1 | 7.2 | 17.6 | 65.8 | 34296.6 |
| Secchi disk (m) | 21 | 3.61 | 3.16 | 0.05 | 1.01 | 2.79 | 5.07 | 16 |
| Chlorophyll-*a* (mg m^-3^) | 13 | 0.01 | 0.02 | 0 | 0 | 0 | 0.01 | 0.14 |
| Dissolved organic carbon (mg m^-3^) | 4 | 7.74 | 14.03 | 0.25 | 1.9 | 4.7 | 9.2 | 208 |
| Maximum depth (m) | 12 | 17.07 | 23.5 | 0.2 | 3.65 | 9.8 | 21.35 | 200 |
| Forestry (% catchment) | 0 | 7.63 | 18.02 | 0 | 0 | 0.21 | 5.21 | 97.76 |
| Native (% catchment) | 0 | 38.04 | 37.15 | 0 | 1.79 | 26.69 | 75.98 | 100 |
| Distance to road (m) | 0 | 3225.31 | 5683.76 | 3.92 | 393.12 | 885.57 | 2954.05 | 41474.91 |

Supplementary Material 2. List of putative bacterial pathogens (Order, Family and species) detected in lake sediment and water samples. Pathogen ASVs were filtered from the whole bacterial community dataset based on the multiple bacterial pathogen detection pipeline and database provided by Yang et al. ^37^ available at <https://github.com/LorMeBioAI/MBPD>.

| **Order** | **Family** | **Species** |
| --- | --- | --- |
| Acidobacteriales | Koribacteraceae | *uncultured Acidobacteria bacterium* |
| Acidobacteriales | Koribacteraceae | *uncultured bacterium* |
| Actinomycetales | Actinomycetaceae | *Actinomyces oris* |
| Actinomycetales | Actinomycetaceae | *Actinomyces viscosus* |
| Actinomycetales | Actinomycetaceae | *Schaalia odontolytica* |
| Bacillales | Bacillaceae | *Bacillus cereus* |
| Bacillales | Bacillaceae | *Bacillus cytotoxicus* |
| Bacillales | Bacillaceae | *Bacillus licheniformis* |
| Bacillales | Bacillaceae | *Bacillus megaterium* |
| Bacillales | Bacillaceae | *Bacillus thuringiensis* |
| Bacillales | Planococcaceae | *Lysinibacillus sphaericus* |
| Bacteroidales | Bacteroidaceae | *Bacteroides vulgatus* |
| Bifidobacteriales | Bifidobacteriaceae | *Gardnerella vaginalis 409-05* |
| Burkholderiales | Alcaligenaceae | *Achromobacter xylosoxidans* |
| Burkholderiales | Alcaligenaceae | *Alcaligenes faecalis* |
| Burkholderiales | Burkholderiaceae | *Ralstonia insidiosa* |
| Burkholderiales | Burkholderiaceae | *Ralstonia pickettii* |
| Burkholderiales | Burkholderiaceae | *uncultured Burkholderiales bacterium* |
| Burkholderiales | Comamonadaceae | *Comamonas testosteroni* |
| Burkholderiales | Comamonadaceae | *Delftia acidovorans* |
| Burkholderiales | Comamonadaceae | *uncultured Burkholderiales bacterium* |
| Burkholderiales | Comamonadaceae | *uncultured bacterium* |
| Burkholderiales | Neisseriaceae | *Neisseria mucosa* |
| Burkholderiales | Oxalobacteraceae | *Janthinobacterium agaricidamnosum* |
| Campylobacterales | Arcobacteraceae | *Arcobacter cryaerophilus* |
| Caulobacterales | Caulobacteraceae | *Brevundimonas diminuta* |
| Caulobacterales | Caulobacteraceae | *Brevundimonas vesicularis* |
| Clostridiales | Clostridiaceae | *Clostridium beijerinckii* |
| Clostridiales | Clostridiaceae | *Clostridium estertheticum subsp. laramiense* |
| Clostridiales | Clostridiaceae | *Clostridium puniceum* |
| Corynebacteriales | Corynebacteriaceae | *Corynebacterium accolens* |
| Corynebacteriales | Corynebacteriaceae | *Corynebacterium afermentans* |
| Corynebacteriales | Corynebacteriaceae | *Corynebacterium kroppenstedtii* |
| Corynebacteriales | Corynebacteriaceae | *Corynebacterium marinum DSM 44953* |
| Corynebacteriales | Corynebacteriaceae | *Corynebacterium propinquum* |
| Corynebacteriales | Mycobacteriaceae | *Mycobacterium avium* |
| Corynebacteriales | Mycobacteriaceae | *Mycobacterium chubuense* |
| Corynebacteriales | Mycobacteriaceae | *Mycobacterium iranicum* |
| Corynebacteriales | Mycobacteriaceae | *Mycobacterium litorale* |
| Corynebacteriales | Mycobacteriaceae | *Mycobacterium marinum* |
| Corynebacteriales | Mycobacteriaceae | *Mycobacterium mucogenicum* |
| Corynebacteriales | Mycobacteriaceae | *Mycobacterium parascrofulaceum* |
| Corynebacteriales | Mycobacteriaceae | *Mycobacterium rhodesiae* |
| Corynebacteriales | Mycobacteriaceae | *Mycobacterium szulgai* |
| Corynebacteriales | Mycobacteriaceae | *Mycobacterium triplex* |
| Corynebacteriales | Mycobacteriaceae | *Mycobacterium vaccae* |
| Corynebacteriales | Mycobacteriaceae | *Mycobacterium vanbaalenii* |
| Corynebacteriales | Nocardiaceae | *Gordonia terrae* |
| Corynebacteriales | Nocardiaceae | *Rhodococcus erythropolis* |
| Corynebacteriales | Nocardiaceae | *Rhodococcus fascians* |
| Cyanobacteriales | Microcystaceae | *Microcystis aeruginosa* |
| Enterobacterales | Aeromonadaceae | *Aeromonas caviae* |
| Enterobacterales | Aeromonadaceae | *Aeromonas encheleia* |
| Enterobacterales | Aeromonadaceae | *Aeromonas hydrophila* |
| Enterobacterales | Aeromonadaceae | *Aeromonas rivipollensis* |
| Enterobacterales | Aeromonadaceae | *Aeromonas salmonicida* |
| Enterobacterales | Aeromonadaceae | *Aeromonas sanarellii* |
| Enterobacterales | Aeromonadaceae | *Aeromonas sobria* |
| Enterobacterales | Aeromonadaceae | *Aeromonas veronii* |
| Enterobacterales | Aeromonadaceae | *Aeromonas veronii bv. sobria* |
| Enterobacterales | Enterobacteriaceae | *Cedecea neteri* |
| Enterobacterales | Enterobacteriaceae | *Citrobacter braakii* |
| Enterobacterales | Enterobacteriaceae | *Citrobacter freundii* |
| Enterobacterales | Enterobacteriaceae | *Citrobacter werkmanii* |
| Enterobacterales | Enterobacteriaceae | *Enterobacter asburiae* |
| Enterobacterales | Enterobacteriaceae | *Enterobacter cloacae* |
| Enterobacterales | Enterobacteriaceae | *Enterobacter hormaechei subsp. steigerwaltii* |
| Enterobacterales | Enterobacteriaceae | *Enterobacter ludwigii* |
| Enterobacterales | Enterobacteriaceae | *Enterobacteriaceae bacterium* |
| Enterobacterales | Enterobacteriaceae | *Escherichia coli* |
| Enterobacterales | Enterobacteriaceae | *Escherichia fergusonii* |
| Enterobacterales | Enterobacteriaceae | *Klebsiella aerogenes* |
| Enterobacterales | Enterobacteriaceae | *Klebsiella oxytoca* |
| Enterobacterales | Enterobacteriaceae | *Klebsiella pneumoniae* |
| Enterobacterales | Enterobacteriaceae | *Leclercia adecarboxylata* |
| Enterobacterales | Enterobacteriaceae | *Lelliottia amnigena* |
| Enterobacterales | Enterobacteriaceae | *Pantoea agglomerans* |
| Enterobacterales | Enterobacteriaceae | *Raoultella ornithinolytica* |
| Enterobacterales | Enterobacteriaceae | *Serratia ureilytica* |
| Enterobacterales | Erwiniaceae | *Enterobacter cloacae* |
| Enterobacterales | Erwiniaceae | *Enterobacteriaceae bacterium endosymbiont of Coelostomidia jenniferae* |
| Enterobacterales | Erwiniaceae | *Erwinia billingiae* |
| Enterobacterales | Erwiniaceae | *Erwinia persicina* |
| Enterobacterales | Erwiniaceae | *Erwinia rhapontici* |
| Enterobacterales | Erwiniaceae | *Pantoea agglomerans* |
| Enterobacterales | Erwiniaceae | *Pantoea ananatis* |
| Enterobacterales | Hafniaceae | *Hafnia alvei* |
| Enterobacterales | Hafniaceae | *Hafnia paralvei* |
| Enterobacterales | Pasteurellaceae | *Haemophilus haemolyticus* |
| Enterobacterales | Pasteurellaceae | *Haemophilus parainfluenzae ATCC 33392* |
| Enterobacterales | Pectobacteriaceae | *Dickeya chrysanthemi* |
| Enterobacterales | Pectobacteriaceae | *Pectobacterium carotovorum* |
| Enterobacterales | Shewanellaceae | *Shewanella putrefaciens* |
| Enterobacterales | Shewanellaceae | *Shewanella putrefaciens 200* |
| Enterobacterales | Yersiniaceae | *Ewingella americana* |
| Enterobacterales | Yersiniaceae | *Rahnella aquatilis* |
| Enterobacterales | Yersiniaceae | *Rahnella aquatilis CIP 78.65 = ATCC 33071* |
| Enterobacterales | Yersiniaceae | *Rouxiella chamberiensis* |
| Enterobacterales | Yersiniaceae | *Serratia fonticola* |
| Enterobacterales | Yersiniaceae | *Serratia liquefaciens* |
| Enterobacterales | Yersiniaceae | *Serratia marcescens* |
| Enterobacterales | Yersiniaceae | *Serratia plymuthica* |
| Enterobacterales | Yersiniaceae | *Serratia proteamaculans* |
| Enterobacterales | Yersiniaceae | *Yersinia aldovae* |
| Enterobacterales | Yersiniaceae | *Yersinia aleksiciae* |
| Enterobacterales | Yersiniaceae | *Yersinia intermedia* |
| Enterobacterales | Yersiniaceae | *Yersinia kristensenii* |
| Enterobacterales | Yersiniaceae | *Yersinia pseudotuberculosis* |
| Enterobacterales | Yersiniaceae | *Yersinia similis* |
| Exiguobacterales | Exiguobacteraceae | *Exiguobacterium sibiricum* |
| Exiguobacterales | Exiguobacteraceae | *Exiguobacterium sibiricum 255-15* |
| Flavobacteriales | Weeksellaceae | *Chryseobacterium indologenes* |
| Flavobacteriales | Weeksellaceae | *Elizabethkingia meningoseptica* |
| Flavobacteriales | Weeksellaceae | *Elizabethkingia miricola* |
| Flavobacteriales | Weeksellaceae | *Empedobacter brevis* |
| Flavobacteriales | Weeksellaceae | *Empedobacter falsenii genomovar 2* |
| Fusobacteriales | Fusobacteriaceae | *Fusobacterium mortiferum* |
| Lactobacillales | Aerococcaceae | *Aerococcus viridans* |
| Lactobacillales | Carnobacteriaceae | *Dolosigranulum pigrum* |
| Lactobacillales | Enterococcaceae | *Enterococcus cecorum* |
| Lactobacillales | Enterococcaceae | *Enterococcus durans* |
| Lactobacillales | Enterococcaceae | *Enterococcus mundtii* |
| Lactobacillales | Lactobacillaceae | *Leuconostoc gelidum* |
| Lactobacillales | Lactobacillaceae | *Leuconostoc lactis* |
| Lactobacillales | Streptococcaceae | *Lactococcus lactis* |
| Lactobacillales | Streptococcaceae | *Leuconostoc pseudomesenteroides* |
| Lactobacillales | Streptococcaceae | *Streptococcus agalactiae* |
| Lactobacillales | Streptococcaceae | *Streptococcus cristatus* |
| Lactobacillales | Streptococcaceae | *Streptococcus equinus* |
| Lactobacillales | Streptococcaceae | *Streptococcus gallolyticus* |
| Lactobacillales | Streptococcaceae | *Streptococcus mitis* |
| Lactobacillales | Streptococcaceae | *Streptococcus mutans* |
| Lactobacillales | Streptococcaceae | *Streptococcus pneumoniae* |
| Lactobacillales | Streptococcaceae | *Streptococcus salivarius* |
| Lactobacillales | Streptococcaceae | *Streptococcus salivarius subsp. thermophilus* |
| Lactobacillales | Streptococcaceae | *Streptococcus sanguinis* |
| Lactobacillales | Streptococcaceae | *Streptococcus suis* |
| Micrococcales | Brevibacteriaceae | *Brevibacterium casei* |
| Micrococcales | Microbacteriaceae | *Clavibacter michiganensis* |
| Micrococcales | Microbacteriaceae | *Curtobacterium flaccumfaciens* |
| Micrococcales | Microbacteriaceae | *Microbacterium maritypicum* |
| Micrococcales | Microbacteriaceae | *Microbacterium oxydans* |
| Micrococcales | Microbacteriaceae | *Microbacterium testaceum* |
| Micrococcales | Microbacteriaceae | *Rathayibacter toxicus* |
| Micrococcales | Micrococcaceae | *Micrococcus luteus* |
| Micrococcales | Micrococcaceae | *Rathayibacter rathayi* |
| Micrococcales | Micrococcaceae | *Rothia dentocariosa* |
| Micrococcales | Micrococcaceae | *Rothia kristinae* |
| Mycoplasmatales | Mycoplasmataceae | *Mycoplasma wenyonii* |
| Mycoplasmatales | Mycoplasmataceae | *Mycoplasma wenyonii str. Massachusetts* |
| Paracaedibacterales | Paracaedibacteraceae | *metagenome* |
| Paracaedibacterales | Paracaedibacteraceae | *uncultured Rickettsiales bacterium* |
| Peptostreptococcales-Tissierellales | Family XI | *Finegoldia magna* |
| Peptostreptococcales-Tissierellales | Peptostreptococcaceae | *Peptostreptococcus stomatis* |
| Propionibacteriales | Propionibacteriaceae | *Cutibacterium granulosum* |
| Pseudomonadales | Halomonadaceae | *Bacillus subtilis* |
| Pseudomonadales | Moraxellaceae | *Acinetobacter baumannii* |
| Pseudomonadales | Moraxellaceae | *Acinetobacter bereziniae* |
| Pseudomonadales | Moraxellaceae | *Acinetobacter bouvetii* |
| Pseudomonadales | Moraxellaceae | *Acinetobacter calcoaceticus* |
| Pseudomonadales | Moraxellaceae | *Acinetobacter guillouiae* |
| Pseudomonadales | Moraxellaceae | *Acinetobacter gyllenbergii* |
| Pseudomonadales | Moraxellaceae | *Acinetobacter haemolyticus* |
| Pseudomonadales | Moraxellaceae | *Acinetobacter johnsonii* |
| Pseudomonadales | Moraxellaceae | *Acinetobacter johnsonii CIP 64.6* |
| Pseudomonadales | Moraxellaceae | *Acinetobacter johnsonii XBB1* |
| Pseudomonadales | Moraxellaceae | *Acinetobacter junii* |
| Pseudomonadales | Moraxellaceae | *Acinetobacter junii CIP 64.5* |
| Pseudomonadales | Moraxellaceae | *Acinetobacter lwoffii* |
| Pseudomonadales | Moraxellaceae | *Acinetobacter oleivorans* |
| Pseudomonadales | Moraxellaceae | *Acinetobacter pittii* |
| Pseudomonadales | Moraxellaceae | *Acinetobacter seifertii* |
| Pseudomonadales | Moraxellaceae | *Acinetobacter soli* |
| Pseudomonadales | Moraxellaceae | *Acinetobacter ursingii* |
| Pseudomonadales | Moraxellaceae | *Bacillus mycoides* |
| Pseudomonadales | Moraxellaceae | *Moraxella osloensis* |
| Pseudomonadales | Moraxellaceae | *Psychrobacter cryohalolentis K5* |
| Pseudomonadales | Pseudomonadaceae | *Kluyvera intermedia* |
| Pseudomonadales | Pseudomonadaceae | *Pseudomonas alcaliphila* |
| Pseudomonadales | Pseudomonadaceae | *Pseudomonas asturiensis* |
| Pseudomonadales | Pseudomonadaceae | *Pseudomonas brassicacearum* |
| Pseudomonadales | Pseudomonadaceae | *Pseudomonas cannabina* |
| Pseudomonadales | Pseudomonadaceae | *Pseudomonas cichorii* |
| Pseudomonadales | Pseudomonadaceae | *Pseudomonas ficuserectae* |
| Pseudomonadales | Pseudomonadaceae | *Pseudomonas fluorescens* |
| Pseudomonadales | Pseudomonadaceae | *Pseudomonas fragi* |
| Pseudomonadales | Pseudomonadaceae | *Pseudomonas frederiksbergensis* |
| Pseudomonadales | Pseudomonadaceae | *Pseudomonas fulva* |
| Pseudomonadales | Pseudomonadaceae | *Pseudomonas fuscovaginae* |
| Pseudomonadales | Pseudomonadaceae | *Pseudomonas gingeri* |
| Pseudomonadales | Pseudomonadaceae | *Pseudomonas graminis* |
| Pseudomonadales | Pseudomonadaceae | *Pseudomonas granadensis* |
| Pseudomonadales | Pseudomonadaceae | *Pseudomonas koreensis* |
| Pseudomonadales | Pseudomonadaceae | *Pseudomonas libanensis* |
| Pseudomonadales | Pseudomonadaceae | *Pseudomonas lundensis* |
| Pseudomonadales | Pseudomonadaceae | *Pseudomonas mandelii* |
| Pseudomonadales | Pseudomonadaceae | *Pseudomonas marginalis pv. marginalis* |
| Pseudomonadales | Pseudomonadaceae | *Pseudomonas mendocina* |
| Pseudomonadales | Pseudomonadaceae | *Pseudomonas migulae* |
| Pseudomonadales | Pseudomonadaceae | *Pseudomonas monteilii* |
| Pseudomonadales | Pseudomonadaceae | *Pseudomonas moraviensis* |
| Pseudomonadales | Pseudomonadaceae | *Pseudomonas oleovorans* |
| Pseudomonadales | Pseudomonadaceae | *Pseudomonas oryzihabitans* |
| Pseudomonadales | Pseudomonadaceae | *Pseudomonas plecoglossicida* |
| Pseudomonadales | Pseudomonadaceae | *Pseudomonas poae* |
| Pseudomonadales | Pseudomonadaceae | *Pseudomonas psychrophila* |
| Pseudomonadales | Pseudomonadaceae | *Pseudomonas psychrotolerans* |
| Pseudomonadales | Pseudomonadaceae | *Pseudomonas putida* |
| Pseudomonadales | Pseudomonadaceae | *Pseudomonas rhizosphaerae* |
| Pseudomonadales | Pseudomonadaceae | *Pseudomonas rhodesiae* |
| Pseudomonadales | Pseudomonadaceae | *Pseudomonas stutzeri* |
| Pseudomonadales | Pseudomonadaceae | *Pseudomonas synxantha* |
| Pseudomonadales | Pseudomonadaceae | *Pseudomonas syringae* |
| Pseudomonadales | Pseudomonadaceae | *Pseudomonas syringae pv. cerasicola* |
| Pseudomonadales | Pseudomonadaceae | *Pseudomonas taetrolens* |
| Pseudomonadales | Pseudomonadaceae | *Pseudomonas tolaasii* |
| Pseudomonadales | Pseudomonadaceae | *Pseudomonas umsongensis* |
| Pseudomonadales | Pseudomonadaceae | *Pseudomonas veronii* |
| Pseudomonadales | Pseudomonadaceae | *Pseudomonas viridiflava* |
| Pseudomonadales | Pseudomonadaceae | *Pseudomonas xanthomarina* |
| Pseudomonadales | Pseudomonadaceae | *Pseudomonas yamanorum* |
| Rhizobiales | Rhizobiaceae | *Agrobacterium larrymoorei* |
| Rhizobiales | Rhizobiaceae | *Agrobacterium radiobacter* |
| Rhizobiales | Rhizobiaceae | *Agrobacterium rubi* |
| Rhizobiales | Stappiaceae | *Pannonibacter phragmitetus* |
| Rhizobiales | Xanthobacteraceae | *Afipia birgiae* |
| Rhizobiales | Xanthobacteraceae | *Afipia broomeae* |
| Rhizobiales | Xanthobacteraceae | *Afipia clevelandensis ATCC 49720* |
| Rhodobacterales | Rhodobacteraceae | *Paracoccus yeei* |
| Rickettsiales | Rickettsiaceae | *uncultured Rickettsiales bacterium* |
| Saccharimonadales | norank | *uncultured Candidatus Saccharibacteria bacterium* |
| Sphingomonadales | Sphingomonadaceae | *Sphingomonas alpina* |
| Sphingomonadales | Sphingomonadaceae | *Sphingomonas hengshuiensis* |
| Sphingomonadales | Sphingomonadaceae | *Sphingomonas insulae* |
| Sphingomonadales | Sphingomonadaceae | *Sphingomonas koreensis* |
| Sphingomonadales | Sphingomonadaceae | *Sphingomonas lutea* |
| Sphingomonadales | Sphingomonadaceae | *Sphingomonas melonis* |
| Sphingomonadales | Sphingomonadaceae | *Sphingomonas panacis* |
| Sphingomonadales | Sphingomonadaceae | *Sphingomonas paucimobilis* |
| Sphingomonadales | Sphingomonadaceae | *Sphingomonas wittichii RW1* |
| Staphylococcales | Gemellaceae | *Gemella haemolysans* |
| Staphylococcales | Gemellaceae | *Gemella sanguinis* |
| Staphylococcales | Staphylococcaceae | *Staphylococcus aureus* |
| Staphylococcales | Staphylococcaceae | *Staphylococcus capitis* |
| Staphylococcales | Staphylococcaceae | *Staphylococcus epidermidis* |
| Staphylococcales | Staphylococcaceae | *Staphylococcus hominis* |
| Staphylococcales | Staphylococcaceae | *Staphylococcus pasteuri* |
| Staphylococcales | Staphylococcaceae | *Staphylococcus sciuri* |
| Staphylococcales | Staphylococcaceae | *Staphylococcus warneri* |
| Streptomycetales | Streptomycetaceae | *Streptomyces albidoflavus* |
| Streptomycetales | Streptomycetaceae | *Streptomyces globisporus* |
| Streptomycetales | Streptomycetaceae | *Streptomyces pratensis* |
| Veillonellales-Selenomonadales | Veillonellaceae | *Veillonella parvula* |
| Xanthomonadales | Xanthomonadaceae | *Stenotrophomonas maltophilia* |
| Xanthomonadales | Xanthomonadaceae | *Stenotrophomonas rhizophila* |
| Xanthomonadales | Xanthomonadaceae | *Xanthomonas albilineans* |

Supplementary Material 2. Summary of mean bacterial ASV richness, pathogen ASV richness, and percentage of pathogen ASVs detected in surface sediment and surface water samples at each lake. Values are averaged across replicate samples (n = 3), with pathogen percentages expressed relative to the total bacterial ASV richness.

| Lake Code | Mean total ASVs Sediment | Mean pathogen ASVs Sediment | Mean percent pathogen ASVs Sediment | Mean total ASVs Water | Mean pathogen ASVs Water | Mean percent pathogen ASVs Water |
| --- | --- | --- | --- | --- | --- | --- |
| 52065 | 1825.3 | 7.7 | 5 |  |  |  |
| 53730 | 1087 | 26.7 | 2.5 | 444.3 | 41.3 | 9.4 |
| 47579 | 1071.3 | 22.7 | 2.2 | 425.7 | 7.3 | 1.7 |
| 54738 | 1711 | 34.7 | 2.1 | 635 | 39.7 | 6.4 |
| 50722 | 916 | 11.5 | 1.4 | 322 | 31 | 9.6 |
| 25994 | 1438.7 | 19.7 | 1.4 | 839.7 | 43 | 5.1 |
| 831 | 2991.7 | 30.3 | 1 | 706.3 | 14.7 | 2.1 |
| 50320 | 2270.7 | 17.7 | 1 |  |  |  |
| 54177 | 2732 | 23 | 0.9 | 623.7 | 32.7 | 5.7 |
| 46959 | 2731 | 14.3 | 0.6 | 722 | 17.7 | 2.5 |
| 54132 | 2397.3 | 13 | 0.5 |  |  |  |
| 50314 | 1116.7 | 5.7 | 0.5 | 660 | 13.3 | 2 |
| 53145 | 3822.7 | 19.7 | 0.5 |  |  |  |
| 841 | 2592.3 | 12.3 | 0.5 |  |  |  |
| 12876 | 1543.3 | 6 | 0.5 | 2603 | 78.7 | 3.1 |
| 36981 | 2354 | 12 | 0.5 | 235.7 | 3.3 | 1.5 |
| 195 | 4008.3 | 17.7 | 0.4 | 622.3 | 4 | 0.6 |
| 53392 | 3971.7 | 16.3 | 0.4 |  |  |  |
| 23691 | 2000.7 | 7.3 | 0.4 | 421.7 | 8.7 | 2.1 |
| 34599 | 1279.7 | 4 | 0.3 |  |  |  |
| 52167 | 4925.3 | 15.7 | 0.3 |  |  |  |
| 29956 | 2827 | 8.7 | 0.3 |  |  |  |
| 47859 | 2089 | 6.3 | 0.3 | 870 | 17.7 | 2.2 |
| 46564 | 2778 | 8 | 0.3 | 299.7 | 3 | 1 |
| 53532 | 2429 | 9 | 0.3 |  |  |  |
| 18934 | 3003.7 | 8 | 0.3 | 716.3 | 36.7 | 5.4 |
| 38955 | 2992.3 | 8 | 0.3 | 457 | 1.2 | 0.3 |
| 13456 | 164 | 0.5 | 0.3 |  |  |  |
| 23690 | 2413.3 | 4.3 | 0.3 | 469.7 | 3 | 0.6 |
| 53338 | 2558.3 | 8.3 | 0.2 | 233.7 | 3 | 1.3 |
| 49186 | 1601 | 3 | 0.2 | 2368.3 | 32.3 | 1.4 |
| 52181 | 3362.7 | 7.7 | 0.2 |  |  |  |
| 47228 | 1415.3 | 3.3 | 0.2 | 338.3 | 6.3 | 1.9 |
| 38974 | 2624 | 5.7 | 0.2 |  |  |  |
| 29250 | 2955 | 6.3 | 0.2 | 330.7 | 18.3 | 5.6 |
| 45514 | 2821.7 | 6.3 | 0.2 | 346.3 | 1 | 0.3 |
| 18719 | 2924.7 | 5.7 | 0.2 |  |  |  |
| 30812 | 2897 | 5.7 | 0.2 |  |  |  |
| 36343 | 1827.7 | 3.7 | 0.2 |  |  |  |
| 14422 | 417.3 | 1 | 0.2 | 457 | 12.3 | 2.7 |
| 13736 | 1111 | 2 | 0.2 |  |  |  |
| 18933 | 4048.3 | 7.7 | 0.2 | 693 | 36.3 | 5.3 |
| 15037 | 1322 | 1.7 | 0.2 | 663.7 | 16.7 | 2.5 |
| 36742 | 1529 | 2.7 | 0.2 |  |  |  |
| 29073 | 3256 | 6 | 0.2 | 311.7 | 22.7 | 7.3 |
| 16222 | 2496.3 | 4.7 | 0.2 | 505.7 | 6.3 | 1.2 |
| 34665 | 1519.3 | 2.3 | 0.2 |  |  |  |
| 29917 | 2425 | 4.3 | 0.2 | 277.7 | 16.3 | 6 |
| 54742 | 3861 | 6.7 | 0.2 |  |  |  |
| 16939 | 3743.7 | 6 | 0.2 | 1599.3 | 49 | 3.1 |
| 44747 | 1244 | 2.3 | 0.2 |  |  |  |
| 39364 | 2691.3 | 4.3 | 0.2 | 729.7 | 25 | 3.4 |
| 13447 | 2804 | 4.7 | 0.2 | 803.3 | 39.3 | 4.9 |
| 14208 | 2485 | 2 | 0.2 |  |  |  |
| 43649 | 2265 | 3.7 | 0.2 | 621 | 21.3 | 3.5 |
| 54038 | 1423 | 2.3 | 0.2 | 635 | 33 | 5.7 |
| 51931 | 2694.3 | 4.7 | 0.2 |  |  |  |
| 29614 | 3916 | 6.3 | 0.2 | 356 | 9 | 2.5 |
| 50401 | 1618.7 | 3 | 0.2 | 565 | 20.3 | 3.5 |
| 53547 | 1281 | 2 | 0.2 | 376.7 | 12 | 3.2 |
| 301 | 2564.3 | 4 | 0.2 |  |  |  |
| 52487 | 3002.3 | 4.7 | 0.2 |  |  |  |
| 19575 | 3156.7 | 4.7 | 0.2 |  |  |  |
| 52064 | 2723.7 | 3.7 | 0.2 |  |  |  |
| 15795 | 2052 | 3 | 0.2 | 411 | 18.7 | 4.6 |
| 25790 | 3262.3 | 3 | 0.2 | 298 | 2 | 0.7 |
| 54655 | 1754.3 | 2.7 | 0.2 | 632.7 | 21.3 | 3.4 |
| 40188 | 1678 | 1.3 | 0.1 | 409 | 3.7 | 0.9 |
| 25789 | 763.3 | 1.3 | 0.1 | 226 | 1 | 0.4 |
| 13437 | 2616.3 | 3.7 | 0.1 |  |  |  |
| 24422 | 2164.3 | 3 | 0.1 |  |  |  |
| 50413 | 3444.7 | 4.7 | 0.1 | 114.7 | 2 | 1.4 |
| 52380 | 3891.7 | 5.7 | 0.1 |  |  |  |
| 52115 | 2168.3 | 3 | 0.1 | 280 | 4 | 1.7 |
| 14071 | 1726.7 | 2.3 | 0.1 |  |  |  |
| 54672 | 2238 | 3 | 0.1 |  |  |  |
| 14426 | 1077 | 1.3 | 0.1 | 1252.7 | 47.3 | 3.8 |
| 37530 | 2289 | 3 | 0.1 | 364.7 | 1.3 | 0.4 |
| 49295 | 1413.3 | 2 | 0.1 | 765 | 35.3 | 5.2 |
| 51958 | 2777 | 3.7 | 0.1 | 294 | 9 | 2.8 |
| 53163 | 3461.7 | 5 | 0.1 | 313.7 | 10.7 | 3.4 |
| 54736 | 982.7 | 1.3 | 0.1 |  |  |  |
| 53753 | 1434 | 1.3 | 0.1 |  |  |  |
| 25972 | 3163 | 4 | 0.1 | 515.7 | 2 | 0.4 |
| 54025 | 2046 | 4 | 0.1 | 591 | 19 | 3.2 |
| 46494 | 1492.7 | 2 | 0.1 | 1019 | 72.3 | 7.2 |
| 53300 | 2381.5 | 3 | 0.1 | 628.7 | 19.7 | 3.1 |
| 38421 | 3043.7 | 3.7 | 0.1 | 424.5 | 2 | 0.4 |
| 48597 | 2333.3 | 3 | 0.1 |  |  |  |
| 511 | 2520.7 | 3 | 0.1 |  |  |  |
| L380.6 | 1147 | 1.3 | 0.1 |  |  |  |
| 29104 | 3670 | 4 | 0.1 |  |  |  |
| 14418 | 1165 | 1.3 | 0.1 | 1231 | 9 | 0.7 |
| 45472 | 4695.7 | 5.7 | 0.1 | 1184 | 41 | 3.4 |
| 24701 | 1769 | 2 | 0.1 | 219.3 | 4 | 1.8 |
| 46493 | 1801 | 2 | 0.1 | 358 | 5.3 | 1.4 |
| 24960 | 977.7 | 1 | 0.1 | 182.3 | 2.7 | 1.5 |
| 37529 | 2489 | 2.7 | 0.1 |  |  |  |
| 51827 | 4029 | 4.3 | 0.1 |  |  |  |
| 28942 | 3959.7 | 4 | 0.1 |  |  |  |
| 26277 | 2095.7 | 2.3 | 0.1 | 1053 | 72.3 | 6.9 |
| 46212 | 2180 | 2.3 | 0.1 | 1496 | 42.7 | 2.9 |
| 18951 | 1980 | 2 | 0.1 | 452.3 | 14 | 3.1 |
| 50269 | 2567.3 | 2.7 | 0.1 |  |  |  |
| 49294 | 1574.7 | 1.7 | 0.1 |  |  |  |
| 46051 | 2428.3 | 2.3 | 0.1 | 433.3 | 10 | 2.3 |
| 36096 | 1903.3 | 2 | 0.1 | 431 | 6.7 | 1.5 |
| 46496 | 1712.3 | 1.7 | 0.1 | 385 | 7 | 1.8 |
| 52117 | 3852.7 | 3.7 | 0.1 |  |  |  |
| 48663 | 3811.3 | 4 | 0.1 | 289.7 | 5 | 1.7 |
| 15907 | 2948.7 | 3 | 0.1 | 266 | 13.3 | 4.8 |
| 40534 | 1868 | 2 | 0.1 |  |  |  |
| 54720 | 2259.3 | 2.3 | 0.1 | 861.7 | 17 | 2 |
| 52177 | 2134.5 | 2 | 0.1 |  |  |  |
| 39297 | 1644.7 | 1.7 | 0.1 | 572 | 17.7 | 3.1 |
| 38502 | 2276.5 | 2.5 | 0.1 |  |  |  |
| 52269 | 4634 | 4.7 | 0.1 |  |  |  |
| 39356 | 1578.7 | 1.7 | 0.1 | 724.7 | 32.3 | 4.4 |
| 13446 | 3617.3 | 3.3 | 0.1 | 1348.3 | 49.7 | 3.7 |
| 52566 | 1387 | 1.3 | 0.1 |  |  |  |
| 39225 | 3451.3 | 2.7 | 0.1 | 373.3 | 1 | 0.3 |
| 16131 | 1546.3 | 1.3 | 0.1 | 571 | 9.3 | 1.6 |
| 22999 | 2959.7 | 3 | 0.1 | 421.3 | 2.3 | 0.5 |
| 48669 | 2037.7 | 2 | 0.1 | 304.7 | 15.7 | 5.3 |
| 53683 | 1523 | 1.3 | 0.1 |  |  |  |
| 15013 | 1391.7 | 1.3 | 0.1 | 1512 | 11.3 | 0.8 |
| 16901 | 2545 | 2.3 | 0.1 |  |  |  |
| 53717 | 1010.7 | 1.3 | 0.1 |  |  |  |
| 41679 | 3459.3 | 2.7 | 0.1 | 2486 | 52.3 | 2.3 |
| 27286 | 1303.3 | 1.3 | 0.1 | 428.3 | 25.7 | 6 |
| 47193 | 1937.7 | 1.7 | 0.1 | 549 | 3 | 0.5 |
| 36285 | 3132.3 | 2.7 | 0.1 | 595.7 | 4.3 | 0.7 |
| 21918 | 2086 | 2.3 | 0.1 |  |  |  |
| 48213 | 3123.3 | 2.7 | 0.1 | 221.7 | 19.3 | 8.8 |
| L380.1 | 4129.7 | 3.7 | 0.1 | 534 | 3 | 0.6 |
| 14179 | 1516 | 1.7 | 0.1 |  |  |  |
| 54731 | 1850.3 | 1.3 | 0.1 | 281.3 | 0.3 | 0.1 |
| 47198 | 3012.5 | 2.5 | 0.1 | 687.3 | 2.3 | 0.3 |
| 29541 | 2997.3 | 3.7 | 0.1 |  |  |  |
| 54088 | 1984 | 1.5 | 0.1 | 1288 | 31 | 2.4 |
| 40071 | 2869.3 | 2 | 0.1 | 472.3 | 1.3 | 0.3 |
| 15020 | 946 | 0.7 | 0.1 | 554 | 6 | 1.1 |
| 50782 | 2056.3 | 1.7 | 0.1 | 1973 | 22.3 | 1.1 |
| 40554 | 2060.3 | 1.7 | 0.1 |  |  |  |
| 52268 | 4285 | 3.3 | 0.1 |  |  |  |
| L380.2 | 1950 | 1.7 | 0.1 | 143 | 7 | 4.9 |
| 47491 | 3278.3 | 2.3 | 0.1 | 873.7 | 14 | 1.6 |
| 39191 | 3573.7 | 2.3 | 0.1 | 293 | 4.7 | 1.6 |
| 49180 | 1805.7 | 1.3 | 0.1 | 1316.7 | 19.3 | 1.5 |
| 52475 | 3889.3 | 3 | 0.1 |  |  |  |
| 8138 | 2940.7 | 2.3 | 0.1 | 896.3 | 35.3 | 3.9 |
| 15034 | 1761 | 1.3 | 0.1 | 1184.3 | 9.7 | 0.8 |
| 25044 | 1824.7 | 1.3 | 0.1 | 1643.3 | 41 | 2.7 |
| 39357 | 1776.7 | 1.3 | 0.1 | 763 | 16.3 | 2.1 |
| 40521 | 3605 | 2.3 | 0.1 |  |  |  |
| 46774 | 1957.3 | 1.3 | 0.1 | 1554 | 50 | 3.3 |
| 229 | 4074.7 | 3 | 0.1 |  |  |  |
| 28543 | 3851.3 | 2.7 | 0.1 |  |  |  |
| 36980 | 2562.7 | 1.7 | 0.1 |  |  |  |
| 54730 | 1430.7 | 1.3 | 0.1 | 504.7 | 0.7 | 0.1 |
| 29596 | 2386.7 | 1.7 | 0.1 | 346.3 | 22.3 | 6.5 |
| 46725 | 2186.7 | 1.7 | 0.1 | 993 | 15.3 | 1.6 |
| 50371 | 2463 | 1.7 | 0.1 | 135.3 | 8.7 | 6 |
| 35422 | 1288 | 1 | 0.1 |  |  |  |
| 48673 | 2443 | 1.7 | 0.1 |  |  |  |
| 1744 | 2617 | 2 | 0.1 |  |  |  |
| 36741 | 1519.3 | 1 | 0.1 |  |  |  |
| 15904 | 3445 | 2.3 | 0.1 | 363 | 4.7 | 1.3 |
| 21450 | 3269.7 | 2 | 0.1 | 623.3 | 5.3 | 0.8 |
| 21433 | 2684.7 | 1.7 | 0.1 | 589.3 | 6.3 | 1.1 |
| 24415 | 4686.7 | 3 | 0.1 | 677.3 | 5.7 | 0.8 |
| 53401 | 3447 | 2.3 | 0.1 | 311 | 13.3 | 4.3 |
| 15038 | 1153.7 | 0.7 | 0.1 | 1576.3 | 16.7 | 1.1 |
| 41293 | 2114 | 1.3 | 0.1 | 380 | 1.7 | 0.4 |
| 54732 | 2064.7 | 1.3 | 0.1 | 261.7 | 0.3 | 0.1 |
| 15007 | 1253.7 | 1 | 0.1 |  |  |  |
| 29644 | 2508 | 1.7 | 0.1 |  |  |  |
| 41305 | 3072.3 | 1.7 | 0.1 | 367.3 | 5.3 | 1.4 |
| 47892 | 3867.3 | 2.3 | 0.1 |  |  |  |
| 39050 | 2751.7 | 1.7 | 0.1 | 549 | 6 | 1.1 |
| 49176 | 2063 | 1.7 | 0.1 |  |  |  |
| 13466 | 3110 | 2 | 0.1 | 794.3 | 14 | 1.8 |
| L380.4 | 1654 | 1 | 0.1 |  |  |  |
| 50345 | 3316 | 2 | 0.1 | 277.3 | 2.3 | 0.6 |
| 46484 | 1743.3 | 1 | 0.1 | 637.3 | 29.3 | 4.6 |
| 53549 | 1854 | 1 | 0.1 | 581 | 2 | 0.3 |
| 17286 | 2280.7 | 1.3 | 0.1 |  |  |  |
| 29568 | 2257.3 | 1.3 | 0.1 |  |  |  |
| 4506 | 3433.3 | 2 | 0.1 | 837.3 | 2 | 0.2 |
| 37534 | 3349.7 | 2 | 0.1 | 359.7 | 0.7 | 0.2 |
| L380.13 | 1038.7 | 0.7 | 0.1 |  |  |  |
| 50335 | 4302.3 | 2.3 | 0.1 | 415.7 | 20.7 | 5 |
| 14405 | 815.7 | 0.7 | 0.1 |  |  |  |
| 24423 | 2396 | 1.3 | 0.1 | 167.3 | 2 | 0.9 |
| 53402 | 3542 | 1.7 | 0.1 |  |  |  |
| 11133 | 1842 | 1 | 0.1 | 516.3 | 1 | 0.2 |
| 32805 | 2869.3 | 1.7 | 0.1 |  |  |  |
| 21926 | 3304 | 1.7 | 0.1 | 332 | 18.7 | 5.5 |
| 30380 | 4219.3 | 2.3 | 0.1 | 280.7 | 2 | 0.7 |
| L380.10 | 2290.3 | 1.3 | 0.1 |  |  |  |
| 50704 | 1634.3 | 1 | 0.1 |  |  |  |
| 21917 | 2055.3 | 1 | 0.1 | 258.7 | 23.3 | 9.1 |
| 39621 | 3505 | 1.7 | 0.1 | 447.7 | 9.3 | 1.9 |
| 24633 | 2611.3 | 1.3 | 0.1 | 486.3 | 5.3 | 1.1 |
| 38665 | 2764.3 | 1.3 | 0 | 419.3 | 5 | 1.1 |
| 15325 | 2716 | 1.3 | 0 | 363.3 | 1.7 | 0.4 |
| 7512 | 2072.7 | 1 | 0 | 429.7 | 15.7 | 3.7 |
| 21759 | 4028.3 | 2 | 0 | 705 | 15.3 | 2.2 |
| 48456 | 2719.7 | 1.3 | 0 |  |  |  |
| 44746 | 2793.3 | 1.3 | 0 |  |  |  |
| 45434 | 2624.3 | 1.3 | 0 | 665 | 8.3 | 1.2 |
| 48447 | 3412 | 1.7 | 0 |  |  |  |
| 45819 | 2653.7 | 1 | 0 |  |  |  |
| 36215 | 2508.3 | 1.7 | 0 | 754.7 | 7 | 0.9 |
| 51754 | 3105 | 2 | 0 |  |  |  |
| 40535 | 1824.3 | 1 | 0 |  |  |  |
| 54039 | 2185.3 | 1 | 0 |  |  |  |
| 46723 | 3165 | 1.3 | 0 | 1038.3 | 32.3 | 3.3 |
| 50373 | 2488.3 | 1 | 0 | 562.7 | 14.3 | 2.5 |
| 46775 | 3970.7 | 2 | 0 | 1063.7 | 36.3 | 3.4 |
| 40590 | 4132.3 | 1.7 | 0 |  |  |  |
| 13467 | 3597.7 | 1.3 | 0 | 439.3 | 5.3 | 1.2 |
| 44599 | 3322.7 | 1.3 | 0 | 734.7 | 7.3 | 1 |
| 48660 | 2336.7 | 1 | 0 | 328 | 5.3 | 1.6 |
| 25248 | 1481.7 | 0.7 | 0 | 381 | 14.3 | 3.8 |
| 15022 | 1644.3 | 0.7 | 0 |  |  |  |
| 47252 | 645 | 0.3 | 0 | 669.7 | 1.7 | 0.3 |
| 50151 | 7431 | 2.7 | 0 | 342 | 10.3 | 2 |
| 50403 | 3249.7 | 1.3 | 0 | 118.7 | 1 | 0.6 |
| 53315 | 2837 | 1.7 | 0 | 1458 | 26 | 1.8 |
| 16722 | 4356.7 | 1.7 | 0 | 441.7 | 13.3 | 2.9 |
| 15312 | 807 | 0.3 | 0 | 183.7 | 7.3 | 4 |
| 32478 | 2506.7 | 1.3 | 0 |  |  |  |
| 23682 | 3710.3 | 1.3 | 0 |  |  |  |
| 4509 | 3763.7 | 1.3 | 0 |  |  |  |
| 38664 | 3059.7 | 1 | 0 | 567 | 1.5 | 0.2 |
| 5042 | 2241.3 | 0.7 | 0 | 903.3 | 19 | 2.1 |
| 21871 | 2969.3 | 1 | 0 |  |  |  |
| 50270 | 2762 | 1 | 0 | 201.3 | 5 | 2.5 |
| 25519 | 2331.3 | 1 | 0 | 429.7 | 2 | 0.3 |
| 39367 | 1340.3 | 0.3 | 0 | 1356 | 36.3 | 2.6 |
| L380.3 | 1506.7 | 0.7 | 0 | 385 | 4 | 1 |
| 53548 | 2279 | 0.7 | 0 | 615 | 5.3 | 0.9 |
| L380.8 | 3573.7 | 1 | 0 |  |  |  |
| 53304 | 1604.3 | 1.3 | 0 |  |  |  |
| L380.14 | 1024.3 | 0.3 | 0 |  |  |  |
| 24511 | 2735.7 | 0.7 | 0 | 631 | 4 | 0.6 |
| 21434 | 3040.3 | 0.7 | 0 | 551.3 | 5 | 0.9 |
| 36314 | 5189 | 1.3 | 0 | 452.3 | 2.7 | 0.6 |
| 49200 | 1660 | 0.7 | 0 | 2260.3 | 76.7 | 3.4 |
| 12830 | 2364 | 1 | 0 | 1167.3 | 50 | 4.3 |
| 14407 | 1325.3 | 0.3 | 0 |  |  |  |
| 54190 | 2548.3 | 0.7 | 0 | 539.7 | 3.7 | 0.7 |
| 24434 | 1425.7 | 0.3 | 0 |  |  |  |
| 49884 | 3107 | 0.7 | 0 |  |  |  |
| 50710 | 1325.7 | 0.3 | 0 |  |  |  |
| 53707 | 3059 | 0.7 | 0 | 524.7 | 1.7 | 0.3 |
| 41306 | 3361 | 0.7 | 0 |  |  |  |
| 14720 | 1247.7 | 0.3 | 0 | 647.7 | 7 | 1.1 |
| 41314 | 1695.3 | 0.3 | 0 | 1481 | 17.3 | 1.2 |
| 14428 | 2533.7 | 0.3 | 0 | 2617.7 | 61 | 2.4 |
| 47860 | 3620.7 | 0.7 | 0 |  |  |  |
| 48216 | 3370 | 0.7 | 0 | 442 | 11 | 2.4 |
| 45686 | 1673.3 | 0.3 | 0 | 427.3 | 6 | 1.4 |
| 46954 | 2031 | 0.3 | 0 | 477 | 12.3 | 2.5 |
| 14290 | 2652 | 0.7 | 0 | 302.3 | 2 | 0.4 |
| 1 | 1748.7 | 0.3 | 0 |  |  |  |
| 24628 | 2534.7 | 0.3 | 0 | 512 | 6.3 | 1.3 |
| 15929 | 2220 | 0.3 | 0 | 466.7 | 5 | 1 |
| 16202 | 1831 | 0.3 | 0 | 500.3 | 4.3 | 0.8 |
| 36979 | 3183.7 | 0.7 | 0 |  |  |  |
| 24620 | 3099.3 | 0.3 | 0 | 310.3 | 3.3 | 1 |
| 47225 | 2234 | 0.3 | 0 | 736 | 5.7 | 0.8 |
| 44391 | 2077.7 | 0.3 | 0 |  |  |  |
| L380.5 | 2263.3 | 0.3 | 0 |  |  |  |
| 18717 | 3707 | 0.3 | 0 | 522.7 | 8.7 | 1.6 |
| 48455 | 2847.7 | 0.3 | 0 | 275.3 | 15.7 | 5.6 |
| 41299 | 5160.3 | 0.3 | 0 |  |  |  |
| 17761 | 4039 | 0.3 | 0 | 493.7 | 15.3 | 2.9 |
| 23681 |  |  |  | 390.7 | 17.7 | 4.5 |
| 46138 |  |  |  | 1401.3 | 45 | 3.3 |
| 45688 |  |  |  | 523.7 | 12.3 | 2.4 |
| 24621 |  |  |  | 688 | 11.3 | 1.7 |
| 48657 |  |  |  | 239.7 | 2.7 | 1.1 |
| 53545 |  |  |  | 577 | 5 | 0.9 |
| 25748 |  |  |  | 392 | 2.7 | 0.7 |
| 7219 |  |  |  | 410.7 | 0.3 | 0.1 |

Supplementary Material 3. Histogram of numbers of number of bacterial pathogenic taxa per lake in a) water and b) sediment samples.


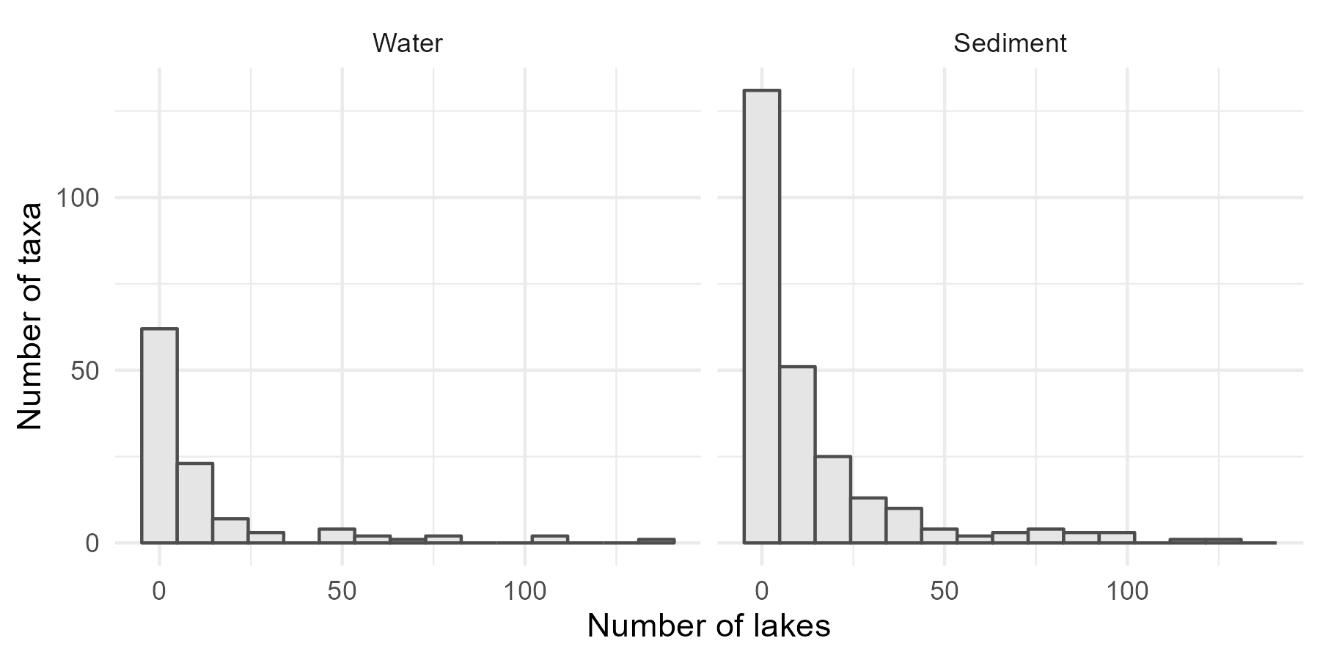


Supplementary Material 4. Results of generalised linear models with negative binomial errors to test the effect of predictor variables on number of bacterial pathogenic taxa in a) water and b) sediment samples.

| 1. Water | estimate | S.E. | statistic | P |
| --- | --- | --- | --- | --- |
| (Intercept) | 3.21 | 0.07 | 48.93 | <0.001 |
| Total Nitrogen | -0.14 | 0.11 | -1.3 | 0.195 |
| Total Phosphorus | 0.08 | 0.09 | 0.99 | 0.321 |
| High-productivity exotic grass | 0.04 | 0.11 | 0.32 | 0.748 |
| Low-productivity grass | 0 | 0.07 | 0.06 | 0.951 |
| Latitude | -0.08 | 0.1 | -0.82 | 0.415 |
| Altitude | 0.24 | 0.12 | 2 | **0.046** |
| Lake area | 0.01 | 0.09 | 0.09 | 0.925 |
| Secchi disk depth | 0.1 | 0.13 | 0.79 | 0.43 |
| Chlorophyll-*a* | -0.11 | 0.08 | -1.37 | 0.172 |
| Dissolved organic carbon | 0.2 | 0.12 | 1.71 | 0.087 |
| Maximum depth | -0.11 | 0.11 | -0.98 | 0.325 |
| Forestry | -0.04 | 0.08 | -0.56 | 0.573 |
| Native | 0.1 | 0.11 | 0.87 | 0.384 |
| Distance to road | -0.01 | 0.09 | -0.07 | 0.945 |
| 1. Sediment | Estimate | S.E. | statistic | P |
| (Intercept) | 5.7 | 0.05 | 34.23 | <0.001 |
| Total Nitrogen | 1.05 | 0.08 | 0.58 | 0.559 |
| Total Phosphorus | 1.06 | 0.07 | 0.87 | 0.384 |
| High-productivity exotic grass | 0.94 | 0.09 | -0.66 | 0.507 |
| Low-productivity grass | 0.95 | 0.06 | -0.84 | 0.403 |
| Latitude | 0.76 | 0.07 | -3.95 | **<0.001** |
| Altitude | 0.81 | 0.09 | -2.4 | **0.016** |
| Lake area | 1 | 0.07 | -0.07 | 0.947 |
| Secchi disk depth | 1.21 | 0.1 | 1.94 | 0.053 |
| Chlorophyll-*a* | 1.06 | 0.06 | 1.04 | 0.3 |
| Dissolved organic carbon | 0.96 | 0.1 | -0.39 | 0.697 |
| Maximum depth | 0.9 | 0.08 | -1.22 | 0.221 |
| Forestry | 0.89 | 0.06 | -1.86 | 0.063 |
| Native | 0.99 | 0.09 | -0.15 | 0.877 |
| Distance to road | 1.03 | 0.07 | 0.47 | 0.637 |

Supplementary Material 5. Results of distance-based redundancy analysis based on Jaccard’s dissimilarities of the bacterial pathogen community showing each predictor variable's effect on the a) water, and b) sediment bacterial pathogen community composition.

| A) Water Community |  | |  | |  | |  | |  |
| --- | --- | --- | --- | --- | --- | --- | --- | --- | --- |
|  | df | | SS | | F | | P | |  |
| Total Nitrogen | 1 | | 3.79 | | 1.44 | | **0.05** | |  |
| Total Phosphorus | 1 | | 2.64 | | 1 | | 0.45 | |  |
| High-productivity exotic grass | 1 | | 6.19 | | 2.35 | | **<0.001** | |  |
| Low-productivity grass | 1 | | 2.74 | | 1.04 | | 0.37 | |  |
| Latitude | 1 | | 3.52 | | 1.33 | | 0.09 | |  |
| Altitude | 1 | | 3.29 | | 1.25 | | 0.13 | |  |
| Lake area | 1 | | 2.6 | | 0.98 | | 0.44 | |  |
| Secchi disk depth | 1 | | 1.91 | | 0.72 | | 0.93 | |  |
| Chlorophyll-*a* | 1 | | 2.73 | | 1.04 | | 0.38 | |  |
| Dissolved organic carbon | 1 | | 3.41 | | 1.29 | | 0.1 | |  |
| Maximum depth | 1 | | 2.34 | | 0.89 | | 0.65 | |  |
| Forestry | 1 | | 1.82 | | 0.69 | | 0.96 | |  |
| Native | 1 | | 2.07 | | 0.78 | | 0.86 | |  |
| Distance to road | 1 | | 2.47 | | 0.94 | | 0.55 | |  |
| Residual | 166 | | 438.15 | |  | |  | |  |
| b) Sediment community | |  | |  | |  | |  | |
|  | | df | | SS | | F | | P | |
| Total Nitrogen | | 1 | | 1.2 | | 0.8 | | 0.71 | |
| Total Phosphorus | | 1 | | 2.99 | | 1.99 | | **0.01** | |
| High-productivity exotic grass | | 1 | | 1.96 | | 1.31 | | 0.16 | |
| Low-productivity grass | | 1 | | 2.87 | | 1.91 | | **0.01** | |
| Latitude | | 1 | | 2.48 | | 1.65 | | 0.06 | |
| Altitude | | 1 | | 1.49 | | 0.99 | | 0.47 | |
| Lake area | | 1 | | 1.66 | | 1.11 | | 0.3 | |
| Secchi disk depth | | 1 | | 2.2 | | 1.46 | | 0.09 | |
| Chlorophyll-*a* | | 1 | | 1.37 | | 0.91 | | 0.56 | |
| Dissolved organic carbon | | 1 | | 1.88 | | 1.25 | | 0.18 | |
| Maximum. depth | | 1 | | 1.08 | | 0.72 | | 0.8 | |
| Forestry | | 1 | | 2.04 | | 1.36 | | 0.12 | |
| Native | | 1 | | 1.46 | | 0.97 | | 0.47 | |
| Distance to road | | 1 | | 1.22 | | 0.81 | | 0.7 | |
| Residual | | 264 | | 396.52 | |  | |  | |
